# Palmar involvement in adult dermatomyositis: A French multicenter cohort

**DOI:** 10.1016/j.jdin.2026.08.020

**Published:** 2026-09-20

**Authors:** Victoire Lippert, Loris Azoyan, Didier Bessis, François Chasset, Diane Kottler, Nicolas Kluger, Emilie Brenaut, Estelle Charvet, Chloé Grolleau, Marie Jachiet, Thibault Mahevas, Jean-David Bouaziz, Charles Cassius, EMSED Group (Groupe d’Étude des Maladies Systémiques en Dermatologie)

**Affiliations:** a Internal Medicine Department, APHP, Hôpital Saint-Antoine, Paris, France; b Dermatology Department, APHP, Hôpital Saint-Antoine, Paris, France; c APHP, Sorbonne Université, INSERM, Institut Pierre Louis d’Epidémiologie et de Santé Publique, Paris, France; d Dermatology Department, Centre Hospitalier Universitaire de Montpellier, Montpellier, France; e Dermatology Department, APHP, Hôpital Tenon, Paris, France; f Dermatology Department, Centre Hospitalier Universitaire de Caen, Caen, France; g Dermatology and Venereology, Aava Medical Centre, Helsinki, Finland; h Department of Dermatology, Tampere University Hospital, Tampere, Finland; i Dermatology Department, APHP, Hôpital Bichat - Claude Bernard, Paris, France; j Dermatology Department, Centre Hospitalier universitaire de Brest, Brest, France; k Université Paris Cité, Institut de Recherche Saint Louis, INSERM U1342, Paris, France

**Keywords:** connective tissue disease, dermatomyositis, MDA5, myositis, palmar papules

Dermatomyositis (DM) is a group of connective tissue diseases affecting the skin, muscles, and lungs. This cohort aimed to assess the relevance of palmar involvement in DM, previously linked to rapidly progressive interstitial lung disease (ILD).^1^ Consecutive adult patients newly diagnosed with definite or probable DM according to European Neuromuscular Centre^2^ criteria were included at 6 French centers from 2021 to 2024. Myositis-specific antibodies were tested systematically at every center by line immunoassay (Euroimmun EUROLINE or D-Tek BlueDiver). Chest high-resolution computed tomography was performed systematically. Palm photographs were scored by consensus of 3 dermatologists blinded to clinical and serologic data. Three features were identified: palmar papules (PP), pulpitis, and mechanic’s hands (MH). PP were defined as discrete erythematous papules and/or macules on the palmar surface (Fig 1), diffuse palmar erythema was not observed, pulpitis as fingertip erythema with or without ulceration (Fig S1, available via Mendeley at https://data.mendeley.com/datasets/j2nr3pc8n4/1), MH as scaling or hyperkeratosis of the digits or thenar/hypothenar eminences (Fig S2, available via Mendeley at https://data.mendeley.com/datasets/j2nr3pc8n4/1). We included 44 patients with a median age of 55 years (IQR, 43.8-65.0 years). Overall, 27 patients (61%) exhibited palmar involvement. PP were present in 19 (43.2%), more often associated with knee and elbow Gottron sign, and tended to present higher ILD prevalence (Table I). All antibodies were represented in the PP group, although anti-MDA5 antibodies were most frequent. Pulpitis was present in 10 (22.7%) patients, associated with less eyelid erythema (20% vs 61.8%; OR, 0.15; 95% CI, 0.03-0.84) and band erythema (10% vs 70.6%; OR, 0.05; 95% CI, 0.01-0.42).

**Fig 1:**
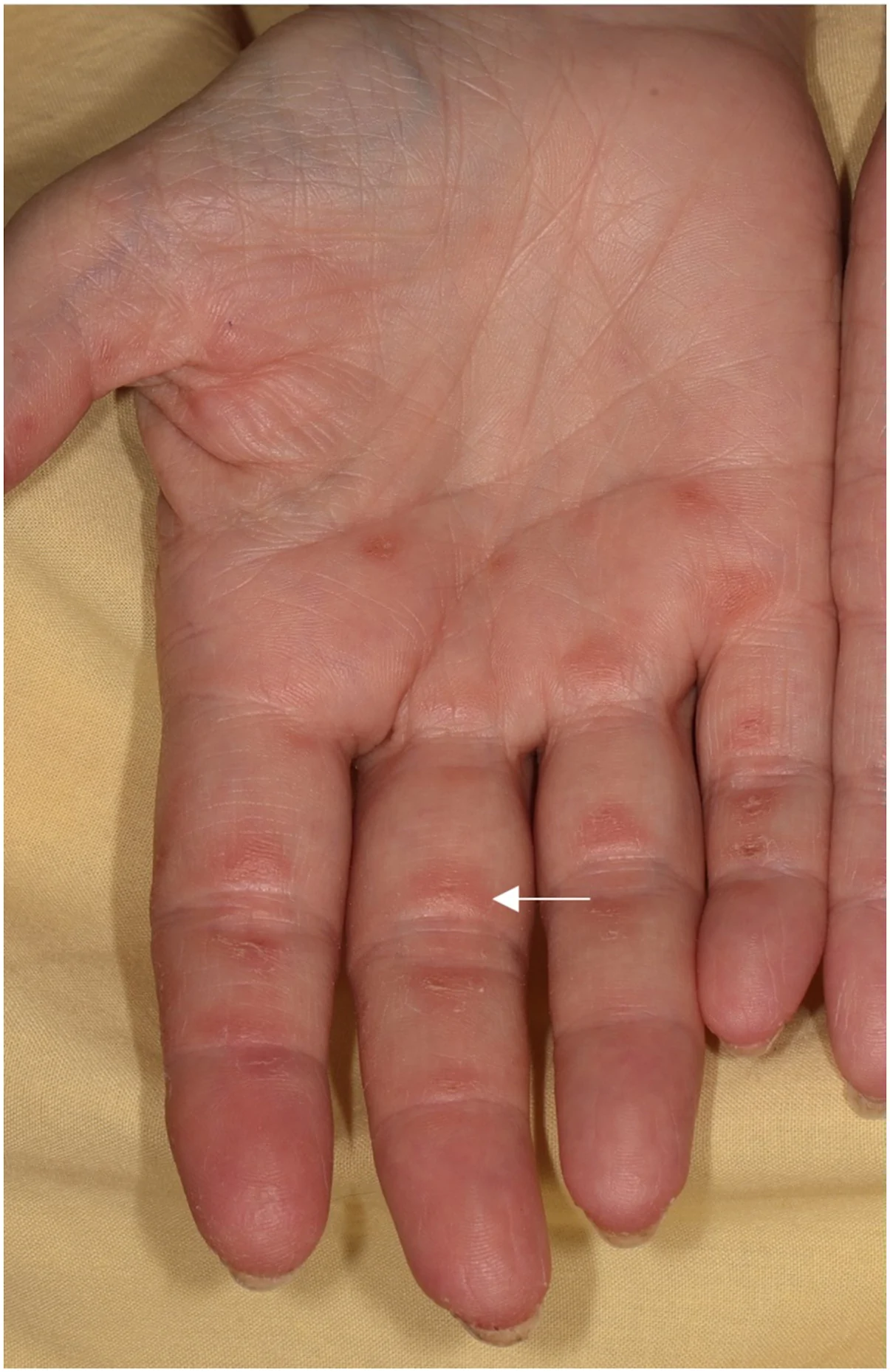
Dermatomyositis palmar papules: representative clinical photograph. Lesions are indicated by white arrows.

**Table I:** Characteristics of patients with DM according to the presence of palmar papules (PP)

Clinic/biology	No PP, *N* = 25	Presence of PP, *N* = 19	OR [95% CI]
Epidemiological characteristics			
Women	18 (72.0)	12 (63.2)	0.67 [0.19-2.39]
Diagnosis age	50.76 [44.1-64.1]	61.68 [43.8-66]	1.00 [0.97-1.04]
Cancer	4 (16.0)	4 (21.1)	1.40 [0.30-6.51]
Skin features			
Eyelids rash	14 (56.0)	9 (47.4)	0.71 [0.21-2.34]
Band erythema	14 (56.0)	11 (57.9)	1.08 [0.32-3.61]
Gottron papules	11 (44.0)	9 (47.4)	1.15 [0.35-3.79]
Mechanic’s hands	4 (16.0)	5 (26.3)	1.88 [0.43-8.22]
Pulpitis	4 (16.0)	6 (31.6)	2.42 [0.57-10.25]
Knees rash	**1 (4.0)**	**6 (31.6)**	**11.08 [1.20-102.20]**
Elbows rash	**3 (12.0)**	**10 (52.6)**	**8.15 [1.81-36.71]**
Muscular involvement			
Amyopathic	15 (60)	6 (31.6)	0.31 [0.09-1.08]
Dysphagia	4 (16.0)	6 (31.6)	2.42 [0.57-10.25]
Pulmonary involvement			
ILD on CT scan	0 (0.0)	5 (26.3)	17.83 [0.92-346.86]∗
Biology			
CK (UI/L) (median [IQR])	196.00 [92.00-721.00]	251.00 [84.00-542.50]	0.99 [0.96-1.03]†
Ferritin, μg/l	136.00 [92.25-195.50]	282.00 [220.00-653.50]	1.04 [0.92-1.17]†
LDH (UI/L)	253.50 [212.8-368.8]	350.50 [291.00-427.25]	1.00 [0.89-1.13]†
Specific myositis antibody			
Seronegative	13 (52.0)	6 (31.6)	0.43 [0.12-1.48]
Anti-Mi2	2 (8.0)	1 (5.3)	0.64 [0.05-7.62]
Anti-NXP2	1 (4.0)	0 (0.0)	0.42 [0.02-10.86]∗
Anti-SAE	4 (16.0)	3 (15.8)	0.98 [0.19-5.04]
MDA5	**1 (4.0)**	**7 (36.8)**	**14.00 [1.54-127.23]**
TIF1γ	4 (16.0)	2 (10.5)	0.62 [0.10-3.79]
Treatment			
IV steroids	**1 (4.0)**	**6 (31.6)**	**14.67 [1.54-139.80]**
IVIg	10 (40.0)	8 (42.1)	1.09 [0.32-3.67]
Methotrexate	**9 (36.0)**	**14 (73.7)**	**4.98 [1.35-18.40]**

Data are presented as n (%) or median [IQR]. Unadjusted ORs with 95% CI.

*CK*, Creatinine phosphokinase; *CT scan*, computed tomography scan; *ILD*, Interstitial lung disease; *IV*, intravenous; *LDH*, lactate dehydrogenase; *MDA5*, melanoma differentiation-associated protein-5; *NXP-2*, nuclear matrix protein-2; *TIF-1 γ*, transcriptional intermediary factor-1γ; *SAE*, small-ubiquitin−like modifier-activating enzyme.

∗ Haldane-Anscombe correction, where a 0 cell occurred.

† ORs for continuous variables were given for a 100-unit increase. Significant values are indicated in bold.

MH occurred in 9 (20%) patients, more frequently associated with anti-MDA5 antibodies (44.4% vs 11.4%; OR, 6.2; 95% CI, 1.16-33.17), higher ferritin levels (OR, 1.35; 95% CI, 1.05-1.75), and lymphopenia (OR, 4.80; 95% CI, 1.00-23.07). Some patients exhibited overlapping phenotypes: 5 presented both PP and MH, and 6 presented both MH and pulpitis.

Our study corroborates that palmar involvement is frequent in DM. Notably, PP (known as inversed Gottron sign) are commonly—not exclusively—associated with anti-MDA5 antibodies, in line with previous studies.^3^, ^4^, ^5^ All 5 ILD cases occurred in anti-MDA5–positive patients; thus, the possible association between PP and ILD cannot be separated from the anti-MDA5 status. MH prevalence in DM varies across studies, from 5% to 50%,^5^ and here it occurred in 20% of patients with elevated ferritin and lymphopenia, consistent with a more inflammatory phenotype. Despite multicenter recruitment and standardized assessment, the limited sample size precluded multivariable analysis. Correction for multiplicity was not applied; the anti-MDA5 association was the prespecified primary comparison but all associations are exploratory, due to low number of patients. The absence of antisyntetase−positive patients, typically associated with MH, likely reflects recruitment mainly from dermatology rather than rheumatology.

In this exploratory cohort, palmar involvement was frequent and associated with distinct clinical and serologic features, including anti-MDA5 status. These findings are hypothesis-generating; validation in larger prospective studies is needed.
